# Shortening Time to Arrival in Out-of-Hospital Cardiac Arrest by Implementing a Dual Dispatch Strategy of EMS and Volunteer Fire Service—A Simulation Study

**DOI:** 10.3390/jcm15072542

**Published:** 2026-03-26

**Authors:** Mathias Maleczek, Jakob Ruthner, Maximilian Scheidl, Christian Fohringer, Bernhard Roessler, Oliver Kimberger

**Affiliations:** 1 Clinical Division of General Anaesthesia and Intensive Care Medicine, Department of Anaesthesia, Intensive Care Medicine and Pain Medicine, Medical University of Vienna, 1090 Vienna, Austria; 2 Academic Simulation Center Vienna, 1090 Vienna, Austria; 3 Fire Department of Bisamberg, 2102 Bisamberg, Austria; 4 Faculty for Medicine, Karl Landsteiner Private University for Health Sciences, 3500 Krems, Austria; 5 Fire Department of Langenzersdorf, 2103 Langenzersdorf, Austria; 6 Notruf Niederoesterreich, 3100 St. Poelten, Austria

**Keywords:** out of hospital cardiac arrest, fire service, simulation

## Abstract

**Background/Objectives**: Survival after out-of-hospital cardiac arrest (OHCA) is strongly influenced by the no-flow interval—the time between cardiac arrest and initiation of cardio-pulmonary resuscitation (CPR)—with the probability of good neurological outcome decreasing by 13% per minute without circulation. Rapid mobilization of all available responders is therefore critical. Fire services, due to their widespread local presence, can shorten response times, but turnout times—particularly in departments staffed with volunteers—may limit their benefit. In sparsely populated regions, dual dispatch of emergency medical service (EMS) and fire services may help reduce arrival times and thus improve outcomes. **Methods**: Response times to 1000 hypothetical OHCAs in Lower Austria (19,000 km^2^, 1.73 million population) were modelled. Travel times were calculated from 121 EMS stations and 1590 fire stations using the fastest route. Turnout times were set at two minutes for EMS and five minutes for fire services, with a sensitivity analysis for eight minutes for fire services. For each event, the shortest travel time was compared for both single EMS and dual EMS and fire service dispatch. **Results**: Mean response time was 10.6 min (SD 4.7) for EMS alone vs. 7.2 min (SD 2.2) with dual dispatch (*p* < 0.0001). At the 90th percentile, times were 16.8 vs. 9.7 min. Within 10 min, 49.0% of cases were reached by EMS alone vs. 92.6% with dual dispatch; fire services arrived first in 62.7% of all simulations. With an 8 min turnout, mean dual-dispatch arrival increased to 8.8 min (SD 2.9), with 68.2% of all patients reached within 10 min and firefighters arriving first in 42.9%. **Conclusions**: Dual dispatch of fire services and EMS significantly reduced response times, particularly in areas with a low population density. Using a dual dispatch strategy, response times were below 10 min in nearly all of the patients.

## 1. Introduction

In cardiac arrest, the length of “no-flow interval”, described as the interval between the onset of cardiac arrest and the initiation of cardiopulmonary resuscitation (CPR), is a crucial parameter for the probability of survival. Early initiation of efficient CPR is strongly associated with improved survival outcomes [1,2,3]. Outcome numbers vary significantly between systems: Approximately 10% of all attempted CPRs resulted in patient survival of at least 30 days, although numbers up to 40% are reported in some cohorts [4,5,6,7,8,9]. The number of patients surviving with good neurological outcomes varies widely from 3 to around 15%, depending on cohort, initial rhythm and the presence of bystander CPR [1,8,10,11].

Although reported values differ among studies, a robust and reproducible association between no-flow interval and patient outcomes has been established, indicating that the likelihood of achieving a favourable neurological outcome decreases by approximately 13% for each additional minute of no-flow interval [1]. No-flow interval has been extensively investigated and can be divided into the following time intervals: dispatch processing time; response time (alarm to curbside arrival); time from curbside arrival to patient contact; and time from patient contact to initiation of cardiopulmonary resuscitation (CPR) or defibrillation [3,12,13,14,15]. The interval from curbside arrival to initiation of CPR is particularly critical, as it may add two to five minutes before life-saving measures are initiated; however, this delay can only be reduced through substantial efforts like facilitating rapid access to buildings or easy access to floors [14,16].

Response time in contrast can be easily reduced by rapid mobilization of all available responders. Such an approach has the potential to significantly decrease no-flow interval and improve patient outcomes [17,18]. Response time consists of two components: turnout time and travel time. Turnout time is defined as time from alarm to departure of the first response vehicle. Travel time is determined primarily by distance, whereas the use of lights and sirens has only a minor effect, particularly in rural areas [19]. Accordingly, dispatching multiple units may reduce travel time.

In rural areas with low population density, EMS faces an additional challenge: the number of ambulance units that must be maintained increases exponentially with stricter response-time targets. Therefore, the dual dispatch of both EMS and fire service warrants further investigation regarding response times and hypothetical influence on patient outcome.

Due to their geographic distribution, fire departments can be a powerful resource for reducing response times and therefore improving outcomes. This has been shown both retrospectively and prospectively [20,21,22,23,24]. Interestingly, while all these trials report shortened times of arrival they had mixed effects on outcome. One study from Switzerland reports mean times of 6 min when additionally dispatching fire brigades compared to 12 min when ambulance services are dispatched alone [20]. The studied area was a small rural area of 190 km^2^. A trial from Houston reported a decrease in response times of 2 min when a fire brigade was dispatched in addition to EMS although fire trucks arrived first at the call location in only 46.7% of cases [24]. This resulted in an estimated probability of return of spontaneous circulation (ROSC) of 20.1%. An important factor of response time is team availability—in volunteer fire departments, turnout time is a relevant factor. A Swedish study reported turnout times from 90 s to five minutes—even in “part-time” firefighters [22].

In many systems, members of volunteer fire departments must first travel from their current location to the fire station, which generally results in longer turnout times compared with professional firefighter units. This raises the question of whether deploying volunteer firefighters to OHCA events can shorten arrival times despite these longer turnout times.

## 2. Materials and Methods

### 2.1. Study Design

A simulation study was conducted to model response times for resuscitation events. As no data from real patients were used, the Ethics Committee of the Medical University of Vienna waived the need for approval on 26 September 2025. The primary objective of the study was to compare arrival-time differences between dispatching EMS alone and dispatching both EMS and fire services to hypothetical OHCA.

The study was conducted using data from Lower Austria, Austria’s largest federal state (aprox. 19,000 km^2^), characterized by a heterogeneous geographic structure that includes extensive rural areas, some mountainous regions, densely populated zones surrounding the metropolitan area of Vienna and five larger cities with >25,000 inhabitants. The total population of lower Austria is 1.73 million people [25]. Most parts of the state consist of sparsely populated countryside, where long travel distances and limited road networks pose inherent challenges for emergency medical services. In contrast, the peri-urban regions around Vienna exhibit higher call volumes but shorter access routes. Importantly, Vienna is not part of Lower Austria and was therefore not part of this simulation.

All data analysis was done using Python 3.8. using the following packages: pandas 2.2.2, scipy 1.13.0, numpy 1.26.4, geopandas 1.1.1 and seaborn 0.13.2 [26,27,28,29,30]. The code is available in Supplement File S1.

### 2.2. Emergency Medical Services

Emergency medical services are provided by three different agencies all dispatched by a central dispatching centre where all emergency calls are answered (Notruf NOE, St. Poelten, Austria). The total number of ambulance stations was collected from the federal dispatch agency (Notruf NOE, St. Poelten, Austria). Of the 125 ambulance stations available, only 121 could be included in our analysis as geolocation was unavailable for 4 of them. Turnout times of EMS are mandated to be two minutes during daytime and three minutes during nighttime. The mean overall turnout time for EMS was 2:09 min in 2025. These time intervals were comparable to internationally reported data. While some urban systems describe turnout times as short as 60 s, rural regions frequently report substantially longer intervals, often exceeding three minutes. This variability reflects differences in system organization, unit deployment strategies, and geographic characteristics [31,32,33,34].

### 2.3. Fire Brigade

Firefighting services in the study region are organized at the municipal level and are mostly staffed with volunteers, who have to leave their workplace or their home, when any operation takes place. All local fire brigades are integrated into a large federal association that issues standards, for example regarding vehicle specifications and the training of firefighting personnel. At the time of this study, firefighting personnel received recurrent basic life support (BLS) lessons, but fire services were not routinely dispatched to resuscitations. BLS-lessons included chest compressions and safe defibrillations as a minimum. There were more than 100,000 active members providing dense coverage across both urban and rural areas. There were 1597 fire stations in Lower Austria of which 1590 could be included as 7 had no available geolocation. Details of EMS and fire station locations can be found in Supplemental Figures S1 and S2.

Turnout times were estimated based on operational experience, supplemented with an analysis of turnout times from comparable German voluntary fire brigades [35]. Fire service turnout times were set to 5 min during daytime and 6 min during nighttime. This is consistent with typical turnout times in Lower Austria. Moreover, unlike standard fire responses, where six to nine firefighters are required to staff a single apparatus, first responder activations for resuscitation typically require a minimum crew of only two persons, which is expected to further reduce turnout times. As both EMS teams and firefighters needed one additional minute during the night, no separate analysis was done for daytime vs. nighttime.

To account for uncertainty in the true turnout time, a sensitivity analysis was conducted using an extended turnout time of 8 min for firefighter volunteers’ response time. This value was selected because it corresponds to the threshold used by the dispatch centre to initiate a second alarm. Additionally, the turnout time threshold at which no difference between the intervention and control groups was observed was determined.

### 2.4. Geographical Data

Geographical data was collected through three main sources: maps were collected from the Federal Statisticians Office (Statistik Austria, Vienna, Austria, CC 4.0 Licence), population data was retrieved from the Global Human Settlement Layer [36] and routes were calculated using the openroutingservice from HeiGit [37]. Travel times were calculated using the fastest route. As the use of lights and sirens typically reduces travel times to a similar extent for both fire and ambulance services, and traffic congestion affects both services comparably, no further adjustments or corrections were applied.

### 2.5. Creating Missions

Because emergencies are not evenly distributed across areas with diverse topographies, the simulated resuscitation events were allocated randomly but weighted by population density. Consequently, population density was incorporated into the random generation of geographic locations for the assumed cardiac arrest cases. Every year, 800–1000 cardiac arrests suitable for additional dispatch occur in Lower Austria. Therefore, 1000 population-weighted random points were created to serve as hypothetical cardiac arrests.

For each location, travel times to the ten nearest fire and EMS stations were calculated to account for spatial variations in distance and travel time while keeping computing resources at a reasonable level. The shortest of these travel times was used for subsequent analyses. To account for turnout times, two minutes were added to EMS travel times and five minutes to fire service travel times.

**Figure 1:**
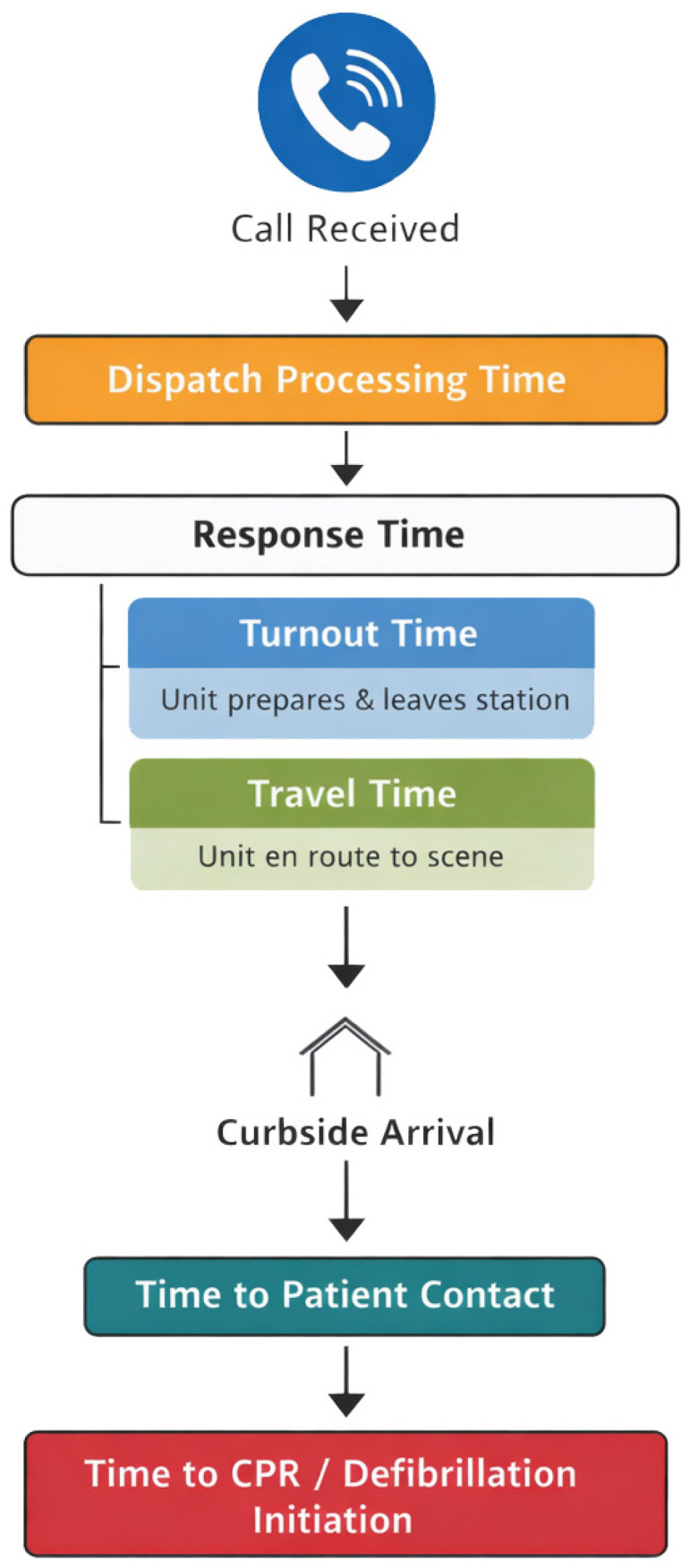
Time intervals: Figure 1 shows the used time intervals. The main goal of this model was to show a possible reduction in travel time even in units with extensive turnout times.

Two response-time measures were then calculated: (1) EMS response time from alarm to arrival at the curb of the scene, and (2) the response time under a dual-dispatch strategy, in which the earliest arrival—either EMS or fire service—was used. Figure 1 shows the used time intervals.

### 2.6. Statistical Analysis

Group characteristics were summarized descriptively. Mean differences between groups were assessed using *t*-tests. A sensitivity analysis was performed in which fire service turnout time was increased to eight minutes while all other parameters remained unchanged. After eight minutes have elapsed, the dispatch centre verifies whether the fire service has left the station. An exploratory analysis was undertaken to determine the turnout time beyond which differences in arrival times were no longer evident.

For secondary outcomes, a generalized linear model was used to examine the effect of population density on arrival times, incorporating both dispatch strategy and population density as predictors.

### 2.7. Use of Artificial Intelligence

Large language models were used solely to improve language and expression in the manuscript text. No content of the manuscript was generated by AI.

## 3. Results

**Figure 2:**
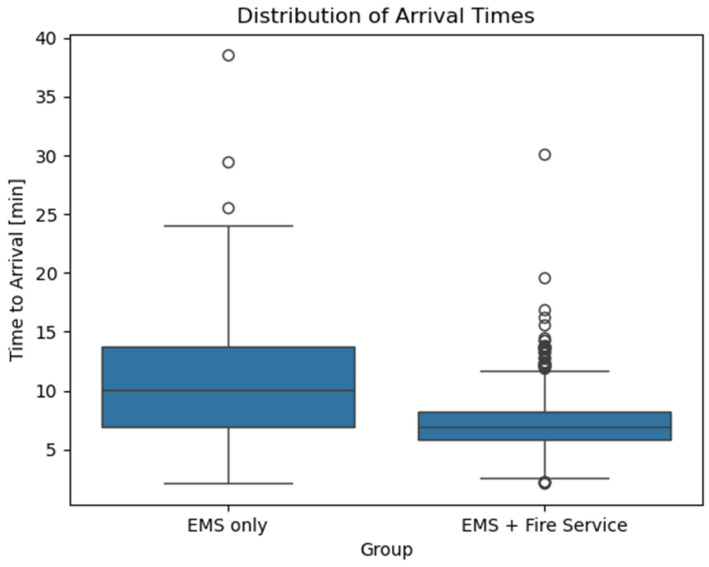
Figure 2 shows the distribution of arrival times between the two groups. Boxplots show medians and interquartile ranges. EMS: Emergency Medical Service.

The mean time to arrival at the curb was 10.6 min (SD: 4.7) in the EMS-only group compared with 7.2 min (SD: 2.2) in the dual-dispatch group, a statistically significant difference (*p* < 0.001, Figure 2).

**Figure 3:**
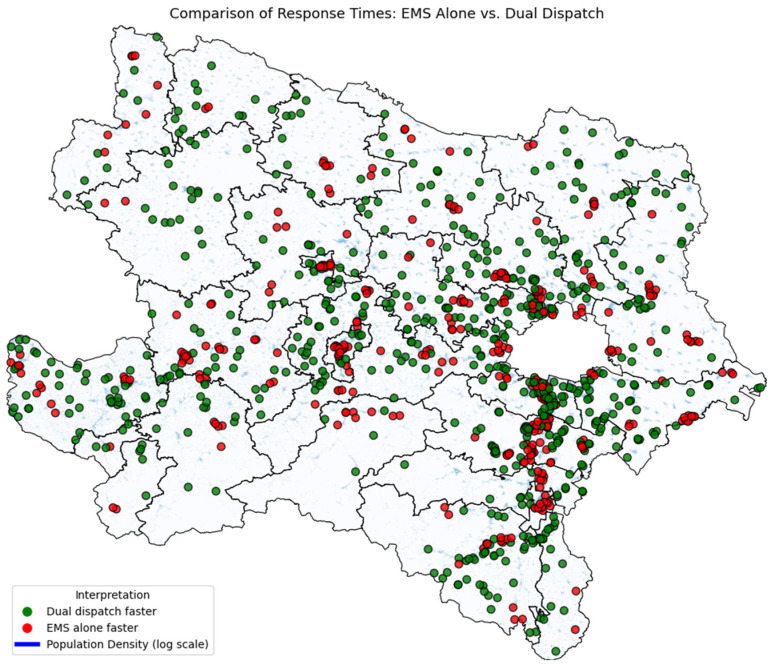
Figure 3 depicts a map of Lower Austria, with each circle representing one simulated cardiac arrest. Red circles indicate cases in which the EMS arrived first, while green circles mark cases where the dual dispatch was faster. The underlying blue shading represents population density on a logarithmic scale. The white spot in the east is the City of Vienna which is a federal state.

This gap was most pronounced at the upper end of the distribution. At the 90th percentile, arrival times were 16.8 min for EMS alone vs. 9.7 min for dual dispatch. Considering a clinically relevant 10 min threshold, 49.0% of missions were reachable within this timeframe in the EMS-only group, compared with 92.6% in the dual-dispatch group. Notably, in 62.7% of cases, the fire service arrived faster than EMS (Figure 3).

**Figure 4:**
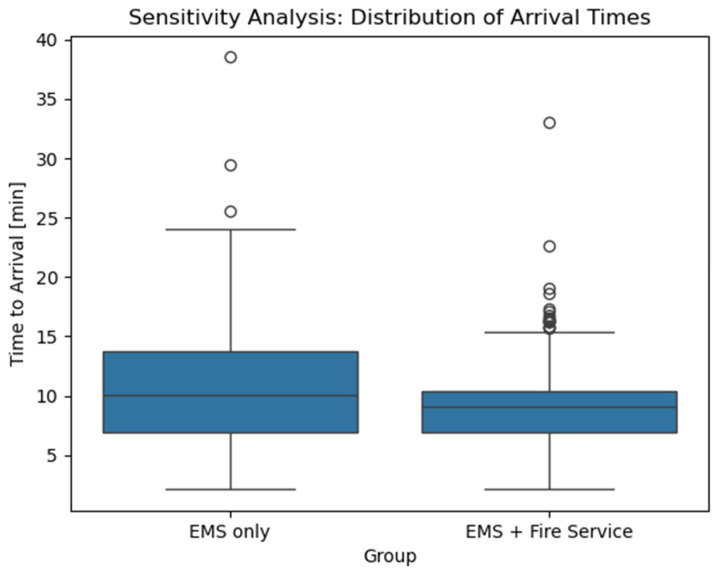
Figure 4 shows the distribution of arrival times between the two groups when 8 min of turnout time were used. Boxplots show medians and interquartile ranges. EMS: Emergency Medical Service.

In a sensitivity analysis assuming an 8 min turnout time for fire services, differences remained significant (*p* < 0.001, Figure 4), although mean arrival time in the dual dispatch group increased to 8.8 min (SD = 2.9), reducing the absolute difference to 1.8 min. Arrival times at the 90th percentile increased to 11.8 min, compared with 16.8 min for EMS alone. Despite this increase, the fire service still arrived earlier at the curb in 42.9% of cases. Within 10 min, 68.2% of missions were reached when EMS and fire services responded together, compared with only 49.0% for EMS alone.

When fire service’s turnout times were extended to 20 min, the arrival times of EMS and the fire service no longer differed significantly.

In a generalized linear model including population density, dispatch type, and their interaction, all predictors were significant (R^2^ = 0.252). Higher population density independently predicted a slightly faster response (β = −0.0009, *p* < 0.001), whereas a positive interaction between population density and dual dispatch strategy indicated that the advantage of dual dispatch decreases modestly as population density increases (β = 0.0006, *p* < 0.001). For example, in a rural area with 100 inhabitants per km^2^, dual EMS–fire service dispatch reduced arrival time from 12.1 to 7.6 min (−4.5 min). In contrast, in a densely populated area with 10,000 inhabitants per km^2^, dual dispatch reduced arrival time from 11.6 to 8.2 min (−3.4 min), illustrating that the relative benefit of dual dispatch is most pronounced in low-density settings.

## 4. Discussion

Dispatching fire service additionally to EMS led to a mean reduction of 3.4 min compared to dispatching EMS alone when using 1000 simulated cardiac arrest cases weighted by population density in a large area of mixed geographic characteristics.

This study illustrates the potential improvements achievable by dispatching fire services in addition to EMS. While previous studies have demonstrated that dual-dispatch strategies can improve patient outcomes, these investigations were conducted primarily in geographically small areas, such as cities or compact districts [20,21,22,24]. This was the first study to evaluate the possible effects of dispatching voluntary fire services in addition to EMS in an entire federal state of 19,000 km^2^ and roughly 1.7 million inhabitants-especially in the context of longer turnout times for fire service than EMS.

In contrast to full-time firefighters, volunteer firefighters needed to travel to the fire station, change clothes and then travel to the point of cardiac arrest. This posed a relevant delay questioning the effectiveness of dispatching fire services. In a Swiss study, dispatching volunteer fire services resulted in improved outcomes although no information about turnout times was provided. Nevertheless, the authors reported that the shortest interval between call and arrival on scene was less than three minutes [20]. This indicated that a number of fire fighters were actually at the fire station at the time of alarm. In the Austrian system most of the voluntary firefighter stations are not manned 24/7. Although some of them, especially in bigger cities, have crews on site during daytime and nighttime, the majority of firefighters need to travel to the fire department station. Consequently, a turnout time of five minutes was used—a time interval based on operational experience and the fact that the number of firefighters needed to respond to a first responder mission can be limited to two persons. As this introduced a reasonable amount of bias, a sensitivity analysis using eight minutes of turnout time was conducted which resulted in a decreased but still relevant reduction in response time.

The limited availability of real-world turnout time data precluded the use of time-dependent models stratified by time of day or day of week. Turnout times may, however, be shorter during nighttime hours, when volunteer firefighters who commute for work are more likely to be present in their home communities. Despite this, the substantial number and geographic distribution of fire stations throughout the region still allow for meaningful reductions in arrival time, even under this extended turnout assumption.

The next logical question concerned the amount of turnout time at which the difference between groups was no longer significant. Although this analysis was strictly exploratory and no correction for multiple testing was applied, we observed that at a turnout time of 20 min, the difference between groups was no longer evident.

A key question concerns the clinical relevance of a 3.4 min reduction in arrival time. It is important to emphasize that a dual-dispatch strategy can only influence the interval between emergency call receipt and arrival at the scene—precisely at the curb of the scene. The time from arrival to initiation of CPR is expected to be largely comparable across responding units and therefore remains unaffected by the intervention. Evidence from Guy et al. demonstrated a 13% decrease in favourable neurological outcome for each additional minute of no-flow interval, underscoring the critical importance of minimizing this interval. However, no-flow interval is not determined solely by time to arrival. It is also substantially influenced by witnessed arrest status, bystander-initiated CPR, phone-assisted CPR, public AED availability, and the presence of trained civilian first responders. These important confounders were not incorporated into the present simulation model [1,3,12,38].

Evidence from regions that have implemented dual-dispatch systems remains heterogeneous. Nordberg et al. reported an increase in 30-day survival from 3.9% to 7.6%, whereas a Slovenian cohort found no significant difference in survival following implementation [21,23].

Survival following OHCA is determined by multiple interconnected factors along the chain of survival; reduction in response time represents only one component of a broader, system-level approach. Data from Japan indicate the existence of a critical response-time threshold: in cases without bystander CPR, neurologically intact survival declines markedly when EMS arrival exceeds 11 min [39]. In the present analysis, the dual-dispatch strategy resulted in arrival at 92% of scenes within ten minutes. These findings suggest that even when volunteers have comparatively long turnout times, their integration into a dual-dispatch model may contribute to clinically meaningful reductions in response intervals. In rural areas characterized by extended travel distances but dense coverage of fire stations, such an approach has the potential to improve survival outcomes.

An alternative to dispatching fire fighters would be the immediate dispatch of lay rescuers to sites of cardiac arrests—for example, using mobile phone apps. This has been shown to effectively improve outcome data [40,41,42]. In regions with low population density, the commonly applied dispatch radius of 350–400 m would only include a small number of potential responders, rendering such short-range activation strategies largely ineffective outside of urban areas. Even within larger towns, arrival times achieved through smartphone-based alerting systems typically ranged from four to five minutes [40,41]. In sparsely populated areas, where travel distances are substantially greater, deployment of fire service vehicles can offset prolonged turnout times compared to using private cars or even travelling by foot. Furthermore, turnout times for cardiac arrest responses may be shorter than those for standard fire service responses. Because only a driver and potentially one additional firefighter are required, crews are not dependent on assembling a full team and may depart immediately upon notification. This operational characteristic may further reduce turnout delays and enhance early response capability.

In Vienna, fire services are dispatched to all cardiac arrests in combination with police additionally to EMS units [43,44,45]. After implementation, survival with good neurological outcome improved in the cohort with witnessed cardiac arrest of cardiac etiology [46]. No significant distribution conflicts concerning the core competencies of the fire services were observed, and firefighting operations were not impeded by the concurrent resuscitation efforts.

The availability of defibrillators may be a limitation. Although many fire services carry automated external defibrillators in their equipment, this is not mandatory, and coverage remains inconsistent since only a basic first aid kit is required on all fire trucks. While it is well known that early defibrillation is essential in shockable OHCA, the existing literature shows that high-quality chest compressions, even in the absence of an immediate defibrillator, can increase the likelihood of successful defibrillation once a device becomes available [38,47,48,49]. Therefore, while equipping all fire apparatus with defibrillators offers clear advantages, even units without them can still contribute to improved patient outcomes through timely initiation of chest compressions making a clinically relevant difference. Additionally, publicly available defibrillators can be incorporated into a system and collected by first responders on their way to scene.

Although this study showed impressive numbers of theoretical improvements in outcomes, some more limitations remain: The analysis was based on a simulated model to estimate the effects of implementing a dual-dispatch strategy, and real-world factors—such as road closures, traffic conditions, or difficulties in locating the patient—may introduce delays that were not reflected in the calculated arrival times. Another limitation concerns the need to ensure that firefighters are convinced that their involvement provides meaningful added value to patient care. While other authors have reported successful implementation of similar systems [20,21,22,23,24], achieving broad acceptance and sustained engagement among volunteer firefighters may still pose a challenge.

Simultaneous missions may occur, and fire units may be dispatched to other operations, potentially limiting availability. Based on data from the Fire Service of Lower Austria, 68,000 missions were recorded in 2025 [50], corresponding to a theoretical mean of 43 missions per station per year (≈1 every 10 days) when evenly distributed across 1590 stations. However, mission volumes are unevenly distributed, with substantially higher call density in urban areas. As these regions generally have well-developed EMS coverage, the projected benefit of our model primarily applies to sparsely populated areas with lower call volumes and more limited EMS resources where simultaneous missions are a rare event.

Another potential limitation is the variability in volunteer turnout and, consequently, dispatch reliability. This challenge is not unique to first-responder missions but also affects the primary responsibility of volunteer fire departments—response to fire incidents. To mitigate this issue, volunteer fire stations typically rely on a very large pool of personnel, ensuring adequate staffing levels even during holidays or vacation periods.

Nevertheless, temporary unavailability of volunteers may influence dispatch performance and could lead to an overestimation of the shown model. Although this risk appears limited within established volunteer systems, it remains an important factor to consider when interpreting the results and when assessing the generalizability of the model.

Existing first responder systems were not incorporated into this simulation due to limited data on their availability and response times. Moreover, the model did not consider the potential occupation of EMS or fire service units with other concurrent missions. Consequently, the effects in either direction may have been obscured, as crews could have been closer to or farther from the simulated incidents than represented in the model.

This simulation study provides a foundation for a prospective, real-world validation to assess potential improvements in patient outcomes. Specifically, reductions in arrival time at the curb are expected to enhance the timeliness of CPR in rural areas with a high density of available first responders, which may ultimately contribute to improved survival.

The code used in this study has been made publicly available in Supplement File S1. An additional module was implemented to retrieve data on EMS and fire services from OpenStreetMap. Analyses based on these OpenStreetMap data produced results that were largely consistent with those obtained using the verified dataset, indicating that this approach can be readily applied in other states or districts.

Post-implementation research will need to show if the estimated numbers can be actually met.

## 5. Conclusions

In this large, simulation-based study of 1000 cardiac arrests across a geographically diverse and sparsely populated region, adding fire service units to EMS dispatch cut response times by more than three minutes—even in a system with five minutes of turnout time for fire services. With dual dispatch, 92% of patients were reached within 10 min, compared with only 49% using EMS alone. These findings highlight the substantial, system-level gains that can be achieved by integrating fire services into early cardiac arrest response.

## Data Availability

Used data is available through the links in Supplement File S1.

## Supplementary Materials

The following supporting information can be downloaded at: https://www.mdpi.com/article/10.3390/jcm15072542/s1, Figure S1: Geographical distribution of fire stations in Lower Austria; Figure S2: Geographical distribution of EMS stations in Lower Austria; Supplement File S1: Used Python Code.

## Abbreviations

The following abbreviations are used in this manuscript:

AED	Automated External Defibrillator
BLS	Basic Life Support
CPR	Cardiopulmonary Resuscitation
EMS	Emergency Medical Service
OHCA	Out-of-Hospital Cardiac Arrest
ROSC	Return of Spontaneous Circulation
SD	Standard Deviation
km^2^	Square Kilometres
aOR	Adjusted Odds Ratio
CI	Confidence Interval
