# Supplementary material for: Shortening Time to Arrival in Out-of-Hospital Cardiac Arrest by Implementing a Dual Dispatch Strategy of EMS and Volunteer Fire Service—A Simulation Study
Source: J Clin Med. 2026 Mar 26;15(7):2542. doi: 10.3390/jcm15072542 (PMC13073219; doi:10.3390/jcm15072542)
Supplement: Supplementary file 1 [file jcm-15-02542-s001.zip › FR Fire Service_code.html]

LowerAustria public


In [ ]:

```
import os
import time
import json
from time import sleep

import numpy as np
import pandas as pd
import scipy.stats as stats
import statsmodels.formula.api as smf

import geopandas as gpd
from shapely.geometry import Point, shape
from shapely.ops import unary_union

import rasterio
import rasterio.mask
from rasterstats import zonal_stats

import openrouteservice
import overpy

import matplotlib.pyplot as plt
import seaborn as sns
from tqdm import tqdm
```

# Base Data¶

## Population data¶

In [ ]:

```
# load Shapefile with districts
shp_path = "STATISTIK_AUSTRIA_POLBEZ_20250101.shp" #get from https://www.data.gv.at/datasets/81f390f9-9b42-3a72-ad9b-e11a4dbebab0?locale=de
gdf = gpd.read_file(shp_path)

# Filter for Lower Austria (g_id 300 - 399)
gdf['g_id'] = pd.to_numeric(gdf['g_id'], errors='coerce')
region = gdf[(gdf["g_id"] >= 300) & (gdf["g_id"] < 400)]

if region.empty:
    raise ValueError("Region not found!")

#Load GHS_POP Raster (EPSG:4326)
raster_path = "GHS_POP_E2030_GLOBE_R2023A_54009_100_V1_0_R4_C20.tif" #https://human-settlement.emergency.copernicus.eu/download.php

with rasterio.open(raster_path) as src:
    print(f"Raster CRS: {src.crs}")

    # reproject regio if necessary
    if region.crs != src.crs:
        region = region.to_crs(src.crs)

    # check overlapping
    if not (
        region.total_bounds[2] < src.bounds.left or
        region.total_bounds[0] > src.bounds.right or
        region.total_bounds[3] < src.bounds.bottom or
        region.total_bounds[1] > src.bounds.top
    ):
        out_image, out_transform = rasterio.mask.mask(src, region.geometry, crop=True)
    else:
        raise ValueError("Region out of bound")

# Maske for values > 0
pop = out_image[0]
masked_pop = np.ma.masked_where(pop <= 0, pop)

# Log-Transform for visualising
log_pop = np.ma.array(np.log1p(masked_pop.data), mask=masked_pop.mask)

# inlcude district boarders
vmax = np.percentile(log_pop.compressed(), 99)

plt.figure(figsize=(10, 8))

# Plot
plt.imshow(
    log_pop,
    cmap="inferno",
    vmax=vmax,
    extent=[
        out_transform[2],  # xmin
        out_transform[2] + log_pop.shape[1] * out_transform[0],  # xmax
        out_transform[5] + log_pop.shape[0] * out_transform[4],  # ymin
        out_transform[5]  # ymax
    ],
    origin="upper"
)

# District boarders as overlay
region.boundary.plot(ax=plt.gca(), edgecolor="cyan", linewidth=1)

plt.colorbar(label="log(1 + population)")
plt.title("Population in Lower Austria (GHS_POP 2020)")
plt.axis("off")
plt.tight_layout()
plt.show()

# Total population for recheck
total_pop = masked_pop.sum()
print(f"Total population GHS_POP in Lower Austria: {int(total_pop):,}")
```

In [ ]:

```
import matplotlib.pyplot as plt
import numpy as np

# mask  invalid values (0 or negativ)
masked_pop = np.ma.masked_where(pop <= 0, pop)

# Modify Contrast
vmax = np.percentile(masked_pop.compressed(), 99)

# Plot
plt.figure(figsize=(10, 8))

# Raster
plt.imshow(
    masked_pop,
    cmap="inferno",
    vmax=vmax,
    extent=[
        out_transform[2],
        out_transform[2] + masked_pop.shape[1] * out_transform[0],
        out_transform[5] + masked_pop.shape[0] * out_transform[4],
        out_transform[5]
    ],
    origin="upper"
)

# District border
region.boundary.plot(ax=plt.gca(), edgecolor="cyan", linewidth=1)

plt.colorbar(label="Population per Raster(linear, vmax = 99. Perzentil)")
plt.title("Population in Lower Austria(GHS_POP 2020)")
plt.axis("off")
plt.tight_layout()
plt.show()

# Total population
total_pop = masked_pop.sum()
print(f"Total population: {int(total_pop):,}")
```

## Calculation of random, weighted coordinates¶

In [ ]:

```
import pandas as pd

# Probability from Raster data
flat_pop = masked_pop.compressed()
flat_indices = np.transpose(np.nonzero(~masked_pop.mask))

# Normalize to sum of 1
prob = flat_pop / flat_pop.sum()

# pick 1000 random pixels
chosen_idx = np.random.choice(len(flat_pop), size=1000, replace=True, p=prob)
chosen_pixel_coords = flat_indices[chosen_idx]

# Transform to coordinates (Lon, Lat) ===
points_lonlat = []
for row, col in chosen_pixel_coords:
    x, y = rasterio.transform.xy(out_transform, row, col, offset='center')
    points_lonlat.append((x, y))

df = pd.DataFrame(points_lonlat, columns=["lon", "lat"])
print(df.head())

# Visualisation
plt.figure(figsize=(10, 8))

# Map
plt.imshow(
    masked_pop,
    cmap="inferno",
    vmax=vmax,
    extent=[
        out_transform[2],
        out_transform[2] + masked_pop.shape[1] * out_transform[0],
        out_transform[5] + masked_pop.shape[0] * out_transform[4],
        out_transform[5]
    ],
    origin="upper"
)

# District borders
region.boundary.plot(ax=plt.gca(), edgecolor="cyan", linewidth=1)

# Add Points
plt.scatter(df["lon"], df["lat"], s=10, c="lime", alpha=0.6, label="Zufällige Punkte")

plt.colorbar(label="Bevölkerung pro Zelle")
plt.title("Zufällig gewichtete Punkte nach Bevölkerungsdichte")
plt.axis("off")
plt.legend()
plt.tight_layout()
plt.show()
```

In [ ]:

```
from pyproj import Transformer

# Create a transformer from raster CRS → WGS84
transformer = Transformer.from_crs(src.crs, "EPSG:4326", always_xy=True)

# Apply the transformation to all points
df[["lon", "lat"]] = df.apply(
    lambda row: transformer.transform(row["lon"], row["lat"]),
    axis=1,
    result_type="expand"
)

df.loc[:, 'google_maps'] = (df['lat']).astype(str) +', '+ (df['lon']).astype(str)
df
df_rand_coordinates = df.copy()
```

In [ ]:

```
#df_rand_coordinates.to_pickle('random_koord.pkl')
df_rand_coordinates = pd.read_pickle('random_koord.pkl')
df_rand_coordinates
```

## Fire and EMS Data¶

## Get all fire stations¶

In [ ]:

```
import overpy
import pandas as pd

# Overpass-API init
api = overpy.Overpass()

# get fire stations
query = """
area["boundary"="administrative"]["name"="Niederösterreich"]["admin_level"="4"]->.searchArea;
(
  node["amenity"="fire_station"](area.searchArea);
  way["amenity"="fire_station"](area.searchArea);
);
out center;
"""
result = api.query(query)


records = []
for node in result.nodes:
    records.append({
        "name": node.tags.get("name", ""),
        "lat": float(node.lat),
        "lon": float(node.lon)
    })
for way in result.ways:
    center = way.center_lat, way.center_lon
    records.append({
        "name": way.tags.get("name", ""),
        "lat": float(center[0]),
        "lon": float(center[1])
    })

df_fire = pd.DataFrame(records)
print(f"Found stations: {len(df_fire)}")
print(df_fire.head())

# CSV export (optional)
df_fire.to_csv("feuerwachen_noe.csv", index=False)
```

Now, this list was compared to the official List of fire stations in Lower Austria. The results are reimported

In [ ]:

```
#Reimport corrected list
df_fire = pd.read_excel('FF_list.xlsx')
df_fire
```

In [ ]:

```
df_fire.loc[df_fire['lat'].isna()]
```

In [ ]:

```
#drop list without location
df_fire = df_fire.dropna()
df_fire
```

In [ ]:

```
import geopandas as gpd
from shapely.geometry import Point

# GeoDataFrame in WGS84 (EPSG:4326)
gdf_fire = gpd.GeoDataFrame(
    df_fire,
    geometry=[Point(xy) for xy in zip(df_fire["lon"], df_fire["lat"])],
    crs="EPSG:4326"
)

# Transform Raster
raster_crs = src.crs
gdf_fire_proj = gdf_fire.to_crs(raster_crs)
```

In [ ]:

```
plt.figure(figsize=(10, 8))
plt.imshow(
    masked_pop,
    cmap="plasma",
    extent=[
        out_transform[2],
        out_transform[2] + masked_pop.shape[1] * out_transform[0],
        out_transform[5] + masked_pop.shape[0] * out_transform[4],
        out_transform[5]
    ],
    origin="upper"
)

# District Boaders
region.boundary.plot(ax=plt.gca(), edgecolor="cyan", linewidth=1)

# Fire stations in same CRS as raster
gdf_fire_proj.plot(ax=plt.gca(), color="red", marker="^", markersize=40, label="Fire Stations")

plt.title("Fire Stations")
plt.axis("off")
plt.legend()
plt.tight_layout()
plt.show()
```

In [ ]:

```
df_fire
```

In [ ]:

```
#rename dfs

coord_df = df_rand_coordinates
fire_df = df_fire
```

In [ ]:

```
coord_df
```

## Get all Ambulance stations¶

ambulance stations were collected from Notruf NÖ

In [ ]:

```
amb_df = pd.read_csv('RK NOE.csv', sep = ';')
amb_df
```

In [ ]:

```
# alternative via OSM:

# Initialize Overpass-API
api = overpy.Overpass()

# Get ambulance stations (admin_level=4)
query = """
area["boundary"="administrative"]["name"="Niederösterreich"]["admin_level"="4"]->.searchArea;
(
  node["emergency"=ambulance_station](area.searchArea);
  way["emergency"=ambulance_station](area.searchArea);
);
out center;
"""

result = api.query(query)

# To DataFrame
records = []
for node in result.nodes:
    records.append({
        "name": node.tags.get("name", ""),
        "lat": float(node.lat),
        "lon": float(node.lon)
    })
for way in result.ways:
    center = way.center_lat, way.center_lon
    records.append({
        "name": way.tags.get("name", ""),
        "lat": float(center[0]),
        "lon": float(center[1])
    })

amb_df = pd.DataFrame(records)
print(f"Found EMS Stations: {len(df_ambulance)}")
print(amb_df.head())
```

In [ ]:

```
amb_df.loc[amb_df.X_GPS.isna()]
```

In [ ]:

```
amb_df = amb_df.dropna(subset = ['X_GPS', 'Y_GPS'])# repair GPS
amb_df['lat'] = (
    amb_df['X_GPS']
    .astype(str)  
    .str.replace('.', '', regex=False)        # remove all existing dots
    .apply(lambda x: float(x[:2] + '.' + x[2:]))  # insert new dot
)
amb_df['lon'] = (
    amb_df['Y_GPS']
    .astype(str)  
    .str.replace('.', '', regex=False)        # remove all existing dots
    .apply(lambda x: float(x[:2] + '.' + x[2:]))  # insert new dot
)
amb_df['BEZ_Ort'] = amb_df[['BEZIRKSSTELLE', 'ORTSSTELLE']].apply(
    lambda x: ' '.join(x.dropna()), axis=1
)

amb_df=amb_df.drop(columns = ['X_GPS', 'Y_GPS', 'X_LAMBERT', 'Y_LAMBERT'])
amb_df
```

In [ ]:

```
import geopandas as gpd
from shapely.geometry import Point

# GeoDataFrame in WGS84 (EPSG:4326)
gdf_amb = gpd.GeoDataFrame(
    amb_df,
    geometry=[Point(xy) for xy in zip(amb_df["lon"], amb_df["lat"])],
    crs="EPSG:4326"
)

# Transform in CRS of Rasters
raster_crs = src.crs
gdf_amb_proj = gdf_amb.to_crs(raster_crs)
```

In [ ]:

```
plt.figure(figsize=(10, 8))
plt.imshow(
    masked_pop,
    cmap="plasma",
    extent=[
        out_transform[2],
        out_transform[2] + masked_pop.shape[1] * out_transform[0],
        out_transform[5] + masked_pop.shape[0] * out_transform[4],
        out_transform[5]
    ],
    origin="upper"
)

# Bezirksgrenzen (projiziert)
region.boundary.plot(ax=plt.gca(), edgecolor="cyan", linewidth=1)

# Feuerwachen im gleichen CRS wie Raster
gdf_amb_proj.plot(ax=plt.gca(), color="red", marker="^", markersize=40, label="EMS Stations")

plt.title("EMS Stations on population map")
plt.axis("off")
plt.legend()
plt.tight_layout()
plt.show()
```

# Routen berechnen¶

## Feuerwehr¶

In [ ]:

```
# === OpenRouteService init ===
client = openrouteservice.Client(key="add your Key")  # https://heigit.org/
```

In [ ]:

```
coord_df
```

In [ ]:

```
fire_df
```

In [ ]:

```
nearest_n = 10           # nearest stations
batch_size_points = 5    # points per matrix
max_routes_per_request = 3000  
batch_size_stations = max(1, max_routes_per_request // len(points_list))


max_retries = 5
pause_between_requests = 1  # seconds

# Initialize result columns
coord_df["min_travel_time"] = np.inf
coord_df["nearest_leitstelle"] = None

# Fire station coordinates
fire_coords = fire_df[["lon", "lat"]].values

# Loop over points in batches
for i in tqdm(range(0, len(coord_df), batch_size_points), desc="Point batches"):
    point_batch = coord_df.iloc[i:i+batch_size_points]
    points_list = point_batch[["lon","lat"]].values.tolist()
    
    # For all points in the batch, determine the nearest N stations
    nearest_wachen_lists = []
    for point in points_list:
        distances = np.linalg.norm(fire_coords - point, axis=1)
        nearest_indices = np.argsort(distances)[:nearest_n]
        nearest_wachen_lists.append(fire_df.iloc[nearest_indices])
    
    # Now create ORS requests per point batch + station batch
    # We proceed in station batches (max batch_size_stations)
    for j in range(0, nearest_n, batch_size_stations):
        # Targets for this station batch
        targets_list = []
        target_indices_per_point = []
        for idx, wachen in enumerate(nearest_wachen_lists):
            wachen_batch = wachen.iloc[j:j+batch_size_stations]
            targets_list.extend(wachen_batch[["lon","lat"]].values.tolist())
            # Keep track of which targets belong to which point
            target_indices_per_point.append((len(targets_list)-len(wachen_batch), len(targets_list)))
        
        # Retry logic for ORS matrix
        for attempt in range(max_retries):
            try:
                matrix = client.distance_matrix(
                    locations = points_list + targets_list,
                    metrics = ['duration'],
                    sources = list(range(len(points_list))),
                    destinations = list(range(len(points_list), len(points_list)+len(targets_list))),
                    profile = 'driving-car'
                )
                break
            except openrouteservice.exceptions.ApiError as e:
                print(f"ORS error for point batch i={i}, attempt {attempt+1}: {e}")
                time.sleep(15)
        else:
            print(f"Point batch i={i}, station batch j={j} failed, skipping")
            continue
        
        # Pause between requests
        time.sleep(pause_between_requests)
        
        durations = np.array(matrix['durations'])  # shape: (len(points_list), len(targets_list))
        
        # Update minimum travel time per point in the batch
        for k, (start_idx, end_idx) in enumerate(target_indices_per_point):
            durs = durations[k, start_idx:end_idx]
            min_idx = np.argmin(durs)
            if durs[min_idx] < coord_df.at[i+k, "min_travel_time"]:
                coord_df.at[i+k, "min_travel_time"] = durs[min_idx]
                coord_df.at[i+k, "nearest_leitstelle"] = nearest_wachen_lists[k].iloc[j + min_idx]["Liste Leitstelle"]

# Check results
coord_df
```

In [ ]:

```
#coord_df.to_pickle('coord_df_fire.pkl')
coord_df = pd.read_pickle('coord_df_fire.pkl')
coord_df
```

In [ ]:

```
coord_df['fire_min_min'] = coord_df['min_travel_time']/60
coord_df
```

In [ ]:

```
coord_df.fire_min_min.hist(bins = 100)
```

## EMS¶

In [ ]:

```
amb_df
```

In [ ]:

```
# Parameters
nearest_n = 10           # Number of nearest stations per point
batch_size_points = 5    # Points per matrix request

max_routes_per_request = 3000  # below 3500, safety buffer
batch_size_stations = max(1, max_routes_per_request // len(points_list))


max_retries = 5
pause_between_requests = 1  # seconds

# Initialize result columns
coord_df["AMB_min_travel_time"] = np.inf
coord_df["AMB_nearest_station"] = None

# Ambulance station coordinates
amb_coords = amb_df[["lon", "lat"]].values

# Loop over points in batches
for i in tqdm(range(0, len(coord_df), batch_size_points), desc="Point batches"):
    point_batch = coord_df.iloc[i:i+batch_size_points]
    points_list = point_batch[["lon","lat"]].values.tolist()
    
    # Determine the nearest N stations for each point in the batch
    nearest_wachen_lists = []
    for point in points_list:
        distances = np.linalg.norm(amb_coords - point, axis=1)
        nearest_indices = np.argsort(distances)[:nearest_n]
        nearest_wachen_lists.append(amb_df.iloc[nearest_indices])
    
    # Now create ORS requests per point batch + station batch
    # We proceed in station batches (max batch_size_stations)
    for j in range(0, nearest_n, batch_size_stations):
        # Targets for this station batch
        targets_list = []
        target_indices_per_point = []
        for idx, wachen in enumerate(nearest_wachen_lists):
            wachen_batch = wachen.iloc[j:j+batch_size_stations]
            targets_list.extend(wachen_batch[["lon","lat"]].values.tolist())
            # Remember which targets belong to which point
            target_indices_per_point.append((len(targets_list)-len(wachen_batch), len(targets_list)))
        
        # Retry logic for ORS matrix
        for attempt in range(max_retries):
            try:
                matrix = client.distance_matrix(
                    locations = points_list + targets_list,
                    metrics = ['duration'],
                    sources = list(range(len(points_list))),
                    destinations = list(range(len(points_list), len(points_list)+len(targets_list))),
                    profile = 'driving-car'
                )
                break
            except openrouteservice.exceptions.ApiError as e:
                print(f"ORS error in point batch i={i}, attempt {attempt+1}: {e}")
                time.sleep(15)
        else:
            print(f"Point batch i={i}, station batch j={j} failed, skipping")
            continue
        
        # Pause between requests
        time.sleep(pause_between_requests)
        
        durations = np.array(matrix['durations'])  # shape: (len(points_list), len(targets_list))
        
        # Update minimum travel time per point in the batch
        for k, (start_idx, end_idx) in enumerate(target_indices_per_point):
            durs = durations[k, start_idx:end_idx]
            min_idx = np.argmin(durs)
            if durs[min_idx] < coord_df.at[i+k, "AMB_min_travel_time"]:
                coord_df.at[i+k, "AMB_min_travel_time"] = durs[min_idx]
                coord_df.at[i+k, "AMB_nearest_station"] = nearest_wachen_lists[k].iloc[j + min_idx]['BEZ_Ort']

# Check results
coord_df
```

In [ ]:

```
#coord_df.to_pickle('coord_df_total.pkl')
coord_df = pd.read_pickle('coord_df_total.pkl')
coord_df
```

In [ ]:

```
coord_df['AMB_min_min'] = coord_df['AMB_min_travel_time']/60
coord_df
```

In [ ]:

```
coord_df.AMB_min_min.hist(bins = 100)
```

In [ ]:

```
coord_df
```

Add turnout times

In [ ]:

```
coord_df.loc[:, 'fire_time_corrected'] = coord_df.loc[:, 'fire_min_min'] + 5
coord_df.loc[:, 'amb_time_corrected'] = coord_df.loc[:, 'AMB_min_min'] + 2
coord_df
```

In [ ]:

```
coord_df.loc[:, 'difference_amb-fire'] = coord_df['amb_time_corrected'] - coord_df['fire_time_corrected']
coord_df
```

In [ ]:

```
coord_df['difference_amb-fire'].hist(bins = 100)
```

# Statistics¶

## Descriptive¶

In [ ]:

```
coord_df
```

### Add Population density¶

In [ ]:

```
# Path to your GeoTIFF
tif_path = "GHS_POP_E2030_GLOBE_R2023A_54009_100_V1_0_R4_C20.tif"

# Your coordinate (Longitude, Latitude)
lon, lat = 15.74, 48.05  # Example: Berlin

# GeoDataFrame containing a point
gdf = gpd.GeoDataFrame(geometry=[Point(lon, lat)], crs="EPSG:4326")

# Load CRS of the raster
with rasterio.open(tif_path) as src:
    raster_crs = src.crs

# Transform point into the CRS of the raster
gdf = gdf.to_crs(raster_crs)

# Query the raster value at the point
with rasterio.open(tif_path) as src:
    row, col = src.index(gdf.geometry.x[0], gdf.geometry.y[0])
    value = src.read(1)[row, col]
print("Population density at the point:", value)

# Buffer of 100 m around the point
buffer = gdf.buffer(100)

# Zonal statistics: mean population density within the buffer
Z_stats = zonal_stats(buffer, tif_path, stats=["mean", "sum"])
print("Mean within 100 m radius:", Z_stats[0]["mean"])
print("Sum within 100 m radius:", Z_stats[0]["sum"])
```

In [ ]:

```
data = coord_df[['lon', 'lat']]

# Path to your GeoTIFF
tif_path = "GHS_POP_E2030_GLOBE_R2023A_54009_100_V1_0_R4_C20.tif"

with rasterio.open(tif_path) as src:
    raster_crs = src.crs
    res_x, res_y = src.res   # Pixel size in the units of the CRS
    # Cell area in km² (only directly correct if the raster uses a meter-based CRS, e.g., EPSG:3035)
    cell_area_km2 = (res_x * res_y) / 1e6  
    
    # Coordinate transformation (WGS84 -> CRS of the raster)
    transformer = Transformer.from_crs("EPSG:4326", raster_crs, always_xy=True)
    
    abs_vals = []
    dens_vals = []
    
    for lon, lat in zip(data["lon"], data["lat"]):
        x, y = transformer.transform(lon, lat)
        row, col = src.index(x, y)
        val = src.read(1)[row, col]
        
        if val == src.nodata:  
            abs_vals.append(None)
            dens_vals.append(None)
        else:
            abs_vals.append(val)                        # absolute population in the cell
            dens_vals.append(val / cell_area_km2)       # inhabitants per km²
    
coord_df["bev_abs"] = abs_vals
coord_df["bev_dichte"] = dens_vals
```

In [ ]:

```
coord_df
```

In [ ]:

```
coord_df.bev_dichte.describe()
```

In [ ]:

```
#coord_df.to_pickle('coord_df_final.pkl')
```

## Primary Outcome¶

In [ ]:

```
coord_df = pd.read_pickle('coord_df_final.pkl')
coord_df
```

In [ ]:

```
coord_df['comb_dur'] = np.where(coord_df['fire_time_corrected'] < coord_df['amb_time_corrected'], coord_df['fire_time_corrected'],  coord_df['amb_time_corrected'])
coord_df
```

In [ ]:

```
stats.ttest_rel(coord_df['amb_time_corrected'], coord_df['comb_dur'])
```

In [ ]:

```
coord_df['amb_time_corrected'].mean()
```

In [ ]:

```
coord_df['amb_time_corrected'].std()
```

In [ ]:

```
coord_df['comb_dur'].mean()
```

In [ ]:

```
coord_df['comb_dur'].std()
```

In [ ]:

```
df_melt = coord_df.reset_index()[['index', 'amb_time_corrected', 'comb_dur']].melt(id_vars = 'index')
df_melt
```

In [ ]:

```
sns.boxplot(data = df_melt, x = 'variable', y = 'value')
plt.ylabel("Time to Arrival [min]")
plt.xlabel("Group")
plt.title("Distribution of Arrival Times")
# Rename group labels
new_labels = {
    'amb_time_corrected': 'EMS only',
    'comb_dur': 'EMS + Fire Service',
}
plt.xticks(ticks=range(len(new_labels)), labels=[new_labels.get(x, x) for x in df_melt['variable'].unique()])
plt.savefig('Figure1_1.png')
plt.show()
```

In [ ]:

```
coord_df[['amb_time_corrected', 'comb_dur']].mean()
```

In [ ]:

```
coord_df[['amb_time_corrected', 'comb_dur']].quantile(q=0.75)
```

In [ ]:

```
coord_df[['amb_time_corrected', 'comb_dur']].quantile(q=0.90)
```

In [ ]:

```
total_points = len(coord_df)
under_10min = (coord_df["comb_dur"] <= 10).sum()

percent_under_10min = (under_10min / total_points) * 100

print(f"{under_10min} out of {total_points} points are reached in under 10 minutes.")
print(f"That is {percent_under_10min:.2f}% of all points.")
```

In [ ]:

```
under_10min_amb = (coord_df["amb_time_corrected"] <= 10).sum()
percent_under_10min_amb = (under_10min_amb / total_points) * 100

print(f"{under_10min_amb} out of {total_points} points are reached by the ambulance service in under 10 minutes.")
print(f"That is {percent_under_10min_amb:.2f}% of all points.")
```

In [ ]:

```
coord_df
```

In [ ]:

```
# number of cases where dual dispatch was faster
(coord_df["comb_dur"] < coord_df["amb_time_corrected"]).sum()
```

In [ ]:

```
df_melt.info()
```

In [ ]:

```
sns.histplot(data = df_melt, x = 'value', hue = 'variable', element = 'step')
plt.legend(title='Group', labels=['EMS + Fire Service', 'EMS alone'])

plt.xlabel("Time to Arrival [Minutes]")
plt.ylabel("Number of Missions")
plt.title("Histogram of Arrival Times")
plt.savefig('Figure2_1.png')
plt.show()
```

## Sensitivity 8min Turnout Time¶

In [ ]:

```
coord_df['fire_time_sens'] = coord_df['fire_time_corrected'] + 3
```

In [ ]:

```
coord_df['comb_dur_sens'] = np.where(coord_df['fire_time_sens'] < coord_df['amb_time_corrected'], coord_df['fire_time_sens'],  coord_df['amb_time_corrected'])
coord_df
```

In [ ]:

```
stats.ttest_rel(coord_df['amb_time_corrected'], coord_df['comb_dur_sens'])
```

In [ ]:

```
coord_df['amb_time_corrected'].mean()
```

In [ ]:

```
coord_df['amb_time_corrected'].std()
```

In [ ]:

```
coord_df['comb_dur_sens'].mean()
```

In [ ]:

```
coord_df['comb_dur_sens'].std()
```

In [ ]:

```
# number of cases where dual dispatch was faster
(coord_df["comb_dur_sens"] < coord_df["amb_time_corrected"]).sum()
```

In [ ]:

```
df_melt = coord_df.reset_index()[['index', 'amb_time_corrected', 'comb_dur_sens']].melt(id_vars = 'index')
df_melt
```

In [ ]:

```
sns.boxplot(data = df_melt, x = 'variable', y = 'value')
plt.ylabel("Time to Arrival [min]")
plt.xlabel("Group")
plt.title("Sensitivity Analysis: Distribution of Arrival Times")
# Rename group labels
new_labels = {
    'amb_time_corrected': 'EMS only',
    'comb_dur_sens': 'EMS + Fire Service',
}
plt.xticks(ticks=range(len(new_labels)), labels=[new_labels.get(x, x) for x in df_melt['variable'].unique()])
plt.savefig('Figure3_1.png')
plt.show()
```

In [ ]:

```
coord_df[['amb_time_corrected', 'comb_dur_sens']].mean()
```

In [ ]:

```
coord_df[['amb_time_corrected', 'comb_dur_sens']].quantile(q=0.75)
```

In [ ]:

```
coord_df[['amb_time_corrected', 'comb_dur_sens']].quantile(q=0.90)
```

In [ ]:

```
total_points = len(coord_df)
under_10min = (coord_df["comb_dur_sens"] <= 10).sum()

percent_under_10min = (under_10min / total_points) * 100

print(f"{under_10min} out of {total_points} points are reached in under 10 minutes.")
print(f"That is {percent_under_10min:.2f}% of all points.")
```

In [ ]:

```
under_10min_amb = (coord_df["amb_time_corrected"] <= 10).sum()
percent_under_10min_amb = (under_10min_amb / total_points) * 100

print(f"{under_10min_amb} out of {total_points} points are reached by the ambulance service in under 10 minutes.")
print(f"That is {percent_under_10min_amb:.2f}% of all points.")
```

In [ ]:

```
df_melt.info()
```

In [ ]:

```
sns.histplot(data = df_melt, x = 'value', hue = 'variable', element = 'step')
plt.legend(title='Group', labels=['EMS + Fire Service', 'EMS alone'])

plt.xlabel("Time to Arrival [Minutes]")
plt.ylabel("Number of Missions")
plt.title("Sensitivity Analysis: Histogram of Arrival Times")
plt.savefig('Figure4_1.png')
plt.show()
```

## Sensitivity max Tournout with no difference¶

In [ ]:

```
coord_df
```

In [ ]:

```
# 5+15 = 20min turnout time
coord_df['fire_time_sensII'] = coord_df['fire_time_corrected'] + 15
```

In [ ]:

```
coord_df['comb_dur_sensII'] = np.where(coord_df['fire_time_sensII'] < coord_df['amb_time_corrected'], coord_df['fire_time_sensII'],  coord_df['amb_time_corrected'])
coord_df
```

In [ ]:

```
stats.ttest_rel(coord_df['amb_time_corrected'], coord_df['comb_dur_sensII'])
```

In [ ]:

```
coord_df['amb_time_corrected'].mean()
```

In [ ]:

```
coord_df['amb_time_corrected'].std()
```

In [ ]:

```
coord_df['comb_dur_sensII'].mean()
```

In [ ]:

```
coord_df['comb_dur_sensII'].std()
```

In [ ]:

```
# number of cases where dual dispatch was faster
(coord_df["comb_dur_sensII"] < coord_df["amb_time_corrected"]).sum()
```

In [ ]:

```
df_melt = coord_df.reset_index()[['index', 'amb_time_corrected', 'comb_dur_sensII']].melt(id_vars = 'index')
df_melt
```

In [ ]:

```
sns.boxplot(data = df_melt, x = 'variable', y = 'value')
plt.ylabel("Time to Arrival [min]")
plt.xlabel("Group")
plt.title("Sensitivity Analysis: Distribution of Arrival Times")
# Rename group labels
new_labels = {
    'amb_time_corrected': 'EMS only',
    'comb_dur_sens': 'EMS + Fire Service',
}
plt.xticks(ticks=range(len(new_labels)), labels=[new_labels.get(x, x) for x in df_melt['variable'].unique()])
plt.savefig('Figure3_1.png')
plt.show()
```

In [ ]:

```
coord_df[['amb_time_corrected', 'comb_dur_sensII']].mean()
```

In [ ]:

```
coord_df[['amb_time_corrected', 'comb_dur_sensII']].quantile(q=0.75)
```

In [ ]:

```
coord_df[['amb_time_corrected', 'comb_dur_sensII']].quantile(q=0.90)
```

In [ ]:

```
total_points = len(coord_df)
under_10min = (coord_df["comb_dur_sensII"] <= 10).sum()

percent_under_10min = (under_10min / total_points) * 100

print(f"{under_10min} out of {total_points} points are reached in under 10 minutes.")
print(f"That is {percent_under_10min:.2f}% of all points.")
```

In [ ]:

```
under_10min_amb = (coord_df["amb_time_corrected"] <= 10).sum()
percent_under_10min_amb = (under_10min_amb / total_points) * 100

print(f"{under_10min_amb} out of {total_points} points are reached by the ambulance service in under 10 minutes.")
print(f"That is {percent_under_10min_amb:.2f}% of all points.")
```

In [ ]:

```
df_melt.info()
```

In [ ]:

```
sns.histplot(data = df_melt, x = 'value', hue = 'variable', element = 'step')
plt.legend(title='Group', labels=['EMS + Fire Service', 'EMS alone'])

plt.xlabel("Time to Arrival [Minutes]")
plt.ylabel("Number of Missions")
plt.title("Sensitivity Analysis: Histogram of Arrival Times")
plt.savefig('Figure4_1.png')
plt.show()
```

## Secondary Outcome¶

In [ ]:

```
df_melt = coord_df.reset_index()[['index', 'amb_time_corrected', 'comb_dur']].melt(id_vars = 'index')
df_melt
```

### Bevölkerungsdichte¶

In [ ]:

```
coord_df
```

In [ ]:

```
sns.scatterplot(data = coord_df.reset_index()[['index', 'amb_time_corrected', 'comb_dur', 'bev_dichte']].melt(id_vars = ['index', 'bev_dichte']), x= 'bev_dichte', y = 'value', hue = 'variable', alpha=0.5)
```

In [ ]:

```
sns.kdeplot(
    data=coord_df.reset_index()[['index', 'amb_time_corrected', 'comb_dur','bev_dichte']].melt(id_vars = ['index', 'bev_dichte']),
    x="bev_dichte",
    y="value",
    hue="variable",
    fill=True
)
```

In [ ]:

```
coord_df
```

In [ ]:

```
import pandas as pd

# Wide → Long
df_long = coord_df.melt(
    id_vars=["bev_dichte"], 
    value_vars=["amb_time_corrected", "comb_dur"],
    var_name="gruppe",
    value_name="dauer"
)

# Dummy-Codierung: Gruppe B vs A
df_long["gruppe"] = (df_long["gruppe"] == "comb_dur").astype(int)

# Regression mit Interaktion
model2 = smf.gls("dauer ~ gruppe * bev_dichte", data=df_long).fit()
print(model2.summary())
```

In [ ]:

```
import seaborn as sns
import matplotlib.pyplot as plt

# Convert data to long format
df_long = coord_df.melt(
    id_vars=["bev_dichte"], 
    value_vars=["amb_time_corrected", "comb_dur"],
    var_name="group",
    value_name="duration"
)

# Rename groups nicely
df_long["group"] = df_long["group"].replace({
    "amb_time_corrected": "Group A (Ambulance)",
    "comb_dur": "Group B (+Fire Department)"
})

# Scatterplot + regression lines
plt.figure(figsize=(12,8))
sns.scatterplot(
    data=df_long, 
    x="bev_dichte", 
    y="duration", 
    hue="group", 
    alpha=0.5
)

sns.regplot(
    data=df_long[df_long["group"]=="Group A (Ambulance)"],
    x="bev_dichte", y="duration", 
    scatter=False, label="Fit A", color="blue"
)

sns.regplot(
    data=df_long[df_long["group"]=="Group B (+Fire Department)"],
    x="bev_dichte", y="duration", 
    scatter=False, label="Fit B", color="orange"
)

plt.title("Duration ~ Population Density (Group A vs. B)")
plt.ylim(bottom=0)
plt.legend()
plt.tight_layout()
plt.show()
```

In [ ]:

```
df_long.dauer.describe()
```

In [ ]:

```
import geopandas as gpd
import rasterio
import rasterio.mask
from shapely.geometry import mapping
import numpy as np
import matplotlib.pyplot as plt
from rasterio.plot import show
from matplotlib.lines import Line2D
import pandas as pd

# Load shapefile ===
shp_path = "STATISTIK_AUSTRIA_POLBEZ_20250101.shp"
gdf = gpd.read_file(shp_path)
gdf['g_id'] = pd.to_numeric(gdf['g_id'], errors='coerce')

# Select a specific region by g_id (e.g., 300-399)
region = gdf[(gdf["g_id"] >= 300) & (gdf["g_id"] < 400)]
if region.empty:
    raise ValueError("Region not found!")

# Open raster and get its CRS ===
raster_path = "GHS_POP_E2030_GLOBE_R2023A_54009_100_V1_0_R4_C20.tif"
with rasterio.open(raster_path) as src:
    raster_crs = src.crs
    pop_data = src.read(1)     # population raster values
    pop_meta = src.meta         # raster metadata

# ransform shapefile to raster CRS ===
region_proj = region.to_crs(raster_crs)

# ask raster to the region ===
with rasterio.open(raster_path) as src:
    region_geom = [mapping(region_proj.unary_union)]
    masked_pop, out_transform = rasterio.mask.mask(src, region_geom, crop=True)
    masked_pop = masked_pop[0]
    masked_pop = np.where(masked_pop < 0, np.nan, masked_pop)  # set invalid values to NaN

# Prepare points ===
# Compute difference in response times: EMS alone vs combined dispatch
coord_df["diff"] = coord_df["amb_time_corrected"] - coord_df["comb_dur"]

# Create GeoDataFrame from points
df_gdf = gpd.GeoDataFrame(
    coord_df,
    geometry=gpd.points_from_xy(coord_df["lon"], coord_df["lat"]),
    crs="EPSG:4326"
)

# Transform points to raster CRS
df_gdf = df_gdf.to_crs(raster_crs)

# Keep only points within the selected region
df_gdf = df_gdf[df_gdf.geometry.within(region_proj.unary_union)]

# Assign colors: Green = Dual dispatch faster, Red = EMS alone faster ===
df_gdf["color"] = np.where(df_gdf["diff"] > 0, "green", "red")

# Plot ===
fig, ax = plt.subplots(figsize=(10, 9))

# Plot population raster (log scale for visibility)
show(np.log1p(masked_pop), transform=out_transform, ax=ax, cmap="Blues", alpha=0.6)

# Plot region boundaries
region_proj.boundary.plot(ax=ax, edgecolor="black", linewidth=0.8)

# Plot points with colors indicating which response is faster
ax.scatter(
    df_gdf.geometry.x,
    df_gdf.geometry.y,
    c=df_gdf["color"],
    edgecolor="black",
    s=50,
    alpha=0.8,
    zorder=3
)

# Create legend
legend_elements = [
    Line2D([0], [0], marker='o', color='w', label='Dual dispatch faster', markerfacecolor='green', markersize=10),
    Line2D([0], [0], marker='o', color='w', label='EMS alone faster', markerfacecolor='red', markersize=10),
    Line2D([0], [0], color='blue', lw=4, label='Population Density (log scale)')
]
ax.legend(handles=legend_elements, title="Interpretation", loc='lower left')

# Set title and remove axes
ax.set_title("Comparison of Response Times: EMS Alone vs. Dual Dispatch", fontsize=13)
ax.set_axis_off()
plt.tight_layout()

# Save figure
plt.savefig('Figure5_1.png')
plt.show()
```

In [ ]:

```
import geopandas as gpd
import rasterio
import rasterio.mask
from shapely.geometry import mapping
import numpy as np
import pandas as pd
import folium
from folium import Choropleth, GeoJson
import matplotlib.pyplot as plt

# Load shapefile ===
shp_path = "STATISTIK_AUSTRIA_POLBEZ_20250101.shp"
gdf = gpd.read_file(shp_path)
gdf["g_id"] = pd.to_numeric(gdf["g_id"], errors="coerce")

# Select a specific region by g_id (e.g., 300-399)
region = gdf[(gdf["g_id"] >= 300) & (gdf["g_id"] < 400)]
if region.empty:
    raise ValueError("Region not found!")

# Open raster and get CRS ===
raster_path = "GHS_POP_E2030_GLOBE_R2023A_54009_100_V1_0_R4_C20.tif"
with rasterio.open(raster_path) as src:
    raster_crs = src.crs
    pop_data = src.read(1)     # population raster values
    pop_meta = src.meta         # raster metadata

# Reproject region and mask raster ===
region_proj = region.to_crs(raster_crs)
with rasterio.open(raster_path) as src:
    region_geom = [mapping(region_proj.unary_union)]
    masked_pop, out_transform = rasterio.mask.mask(src, region_geom, crop=True)
    masked_pop = masked_pop[0]
    masked_pop = np.where(masked_pop < 0, np.nan, masked_pop)

# Prepare points ===
# Compute difference in response times: EMS alone vs combined dispatch
coord_df["diff"] = coord_df["amb_time_corrected"] - coord_df["comb_dur"]

# Create GeoDataFrame for points
df_gdf = gpd.GeoDataFrame(
    coord_df,
    geometry=gpd.points_from_xy(coord_df["lon"], coord_df["lat"]),
    crs="EPSG:4326"
)

# Keep points in WGS84 (needed for web maps)
df_gdf = df_gdf.to_crs("EPSG:4326")

# Define colors ===
# Green = Combined dispatch faster, Red = EMS alone faster
df_gdf["color"] = np.where(df_gdf["diff"] > 0, "green", "red")

# Create interactive map ===
center_lat = df_gdf.geometry.y.mean()
center_lon = df_gdf.geometry.x.mean()

m = folium.Map(location=[center_lat, center_lon], zoom_start=8, tiles="cartodb positron")

# Add points to the map ===
for _, row in df_gdf.iterrows():
    folium.CircleMarker(
        location=[row.geometry.y, row.geometry.x],
        radius=5,
        color=row["color"],
        fill=True,
        fill_color=row["color"],
        fill_opacity=0.8,
        popup=folium.Popup(
            f"<b>Difference:</b> {row['diff']:.2f} min<br>"
            f"<b>Combined dispatch:</b> {row['comb_dur']:.2f} min<br>"
            f"<b>EMS alone:</b> {row['amb_time_corrected']:.2f} min",
            max_width=250
        ),
    ).add_to(m)

#Add district boundaries ===
region_4326 = region.to_crs("EPSG:4326")
GeoJson(
    region_4326,
    name="Districts",
    style_function=lambda x: {
        "color": "black",
        "weight": 1,
        "fillOpacity": 0
    },
).add_to(m)

# Add layer control ===
folium.LayerControl().add_to(m)

# Display / save map ===
m.save("interactive_map.html")
m
```

In [ ]:

```

```

In [ ]:

```

```

In [ ]:

```

```
