# Supplementary material for: Shortening Time to Arrival in Out-of-Hospital Cardiac Arrest by Implementing a Dual Dispatch Strategy of EMS and Volunteer Fire Service—A Simulation Study
Source: J Clin Med. 2026 Mar 26;15(7):2542. doi: 10.3390/jcm15072542 (PMC13073219; doi:10.3390/jcm15072542)
Supplement: Supplementary file 1 [file jcm-15-02542-s001.zip › Supplement.pdf]

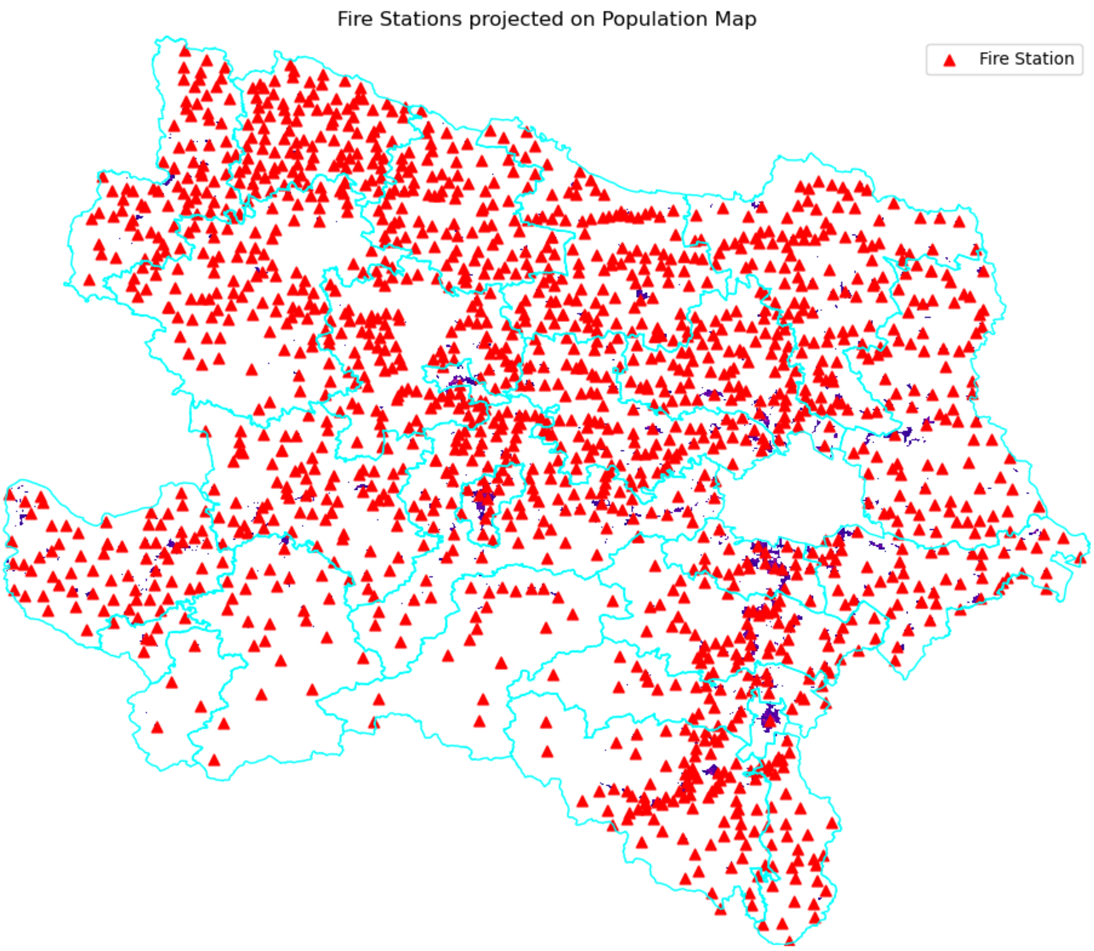

Supplemental Figure S1. Geographical distribution of fire stations in Lower Austria. Population density is indicated by the blue shading, while red triangles mark the locations of individual fire stations.

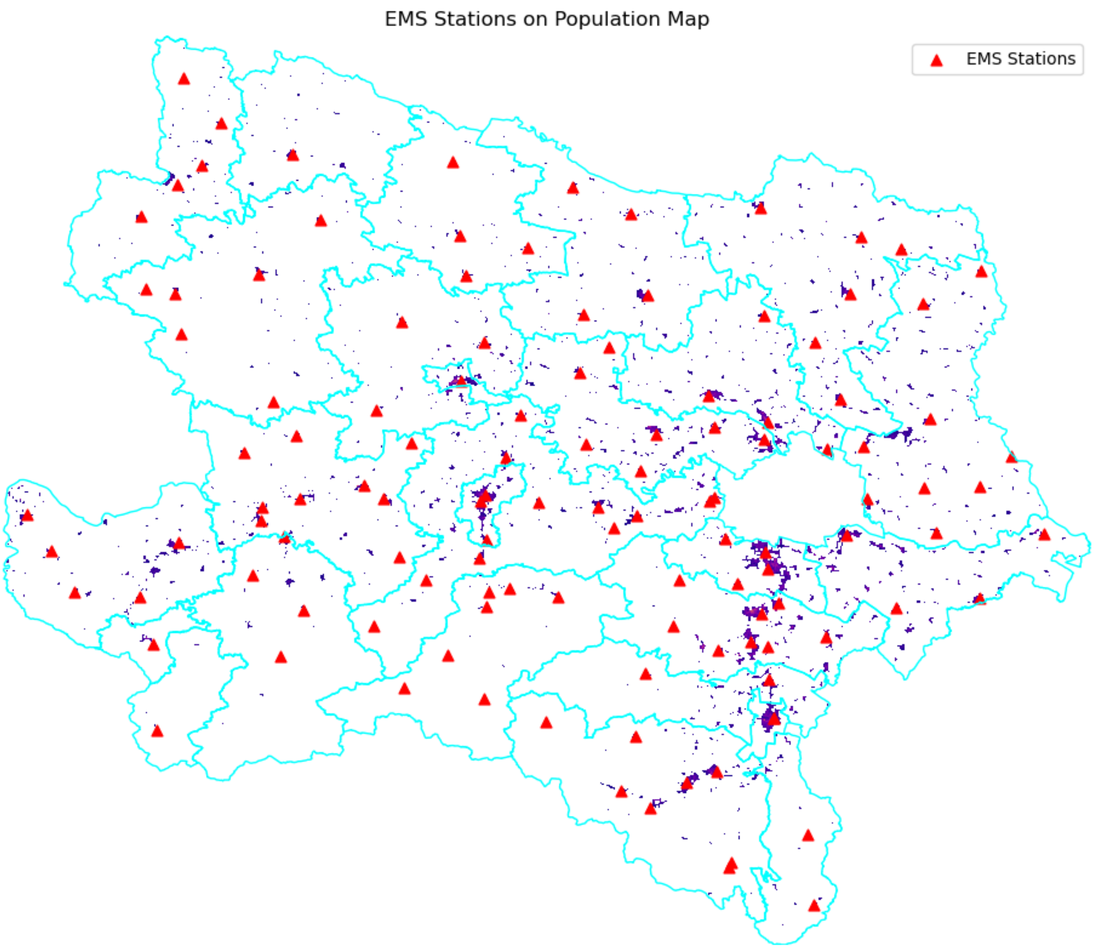

Supplemental Figure S2. Geographical distribution of EMS stations in Lower Austria. Population density is indicated by the blue shading, while red triangles mark the locations of individual EMS stations.
